# Effects of Cyfluthrin Exposure on Neurobehaviour, Hippocampal Tissue and Synaptic Plasticity in Wistar Rats

**DOI:** 10.3390/toxics11120999

**Published:** 2023-12-07

**Authors:** Yongxin Xie, Ji Zhao, Xiaoyu Li, Jian Sun, Huifang Yang

**Affiliations:** 1 School of Public Health, Ningxia Medical University, Yinchuan 750004, China; xieyongxin1991@163.com (Y.X.); zhaosky2021@163.com (J.Z.); lxyandpp@foxmail.com (X.L.); 2 Key Laboratory of Environmental Factors and Chronic Disease Control, No. 1160, Shengli Street, Xingqing District, Yinchuan 750004, China

**Keywords:** cyhalothrin, nerve injury, synaptic plasticity, A_2A_R

## Abstract

This experiment was conducted to study the effects of Cyfluthrin (Cy) exposure on neurobehaviour, hippocampal tissue and synaptic plasticity in Wistar rats. First, it was found that high-dose Cy exposure could cause nerve injury, resulting in symptoms such as deficits in learning and memory ability, spatial exploration and autonomic motor function. Moreover, it was found that medium- and high-dose Cy exposure could cause an abnormal release of the neurotransmitter Glu. Second, brain tissue pathology showed that the middle and high doses of Cy caused tissue deformation, reduced the number of hippocampal puramidal cells, caused a disorder of these cells, decreased the number of Nissl bodies, and caused pyknosis of the hippocampal cell nuclear membrane and serious damage to organelles, indicating that exposure to these doses of Cy may cause hippocampal tissue damage in rats. Third, as the exposure dose increased, morphological changes in hippocampal synapses, including blurred synaptic spaces, a decreased number of synaptic vesicles and a decreased number of synapses, became more obvious. Moreover, the expression levels of the key synaptic proteins PSD-95 and SYP also decreased in a dose-dependent manner, indicating obvious synaptic damage. Finally, the study found that medium and high doses of Cy could upregulate the expression of A_2A_R in the hippocampus and that the expression levels of inflammatory factors and apoptosis-related proteins increased in a dose-dependent manner. Moreover, the expression of A_2A_R mRNA was correlated with neurobehavioural indicators and the levels of inflammatory factors, synaptic plasticity-related factors and apoptosis-related factors, suggesting that Cy may cause nerve damage in rats and that this effect is closely related to A_2A_R.

## 1. Background

Although pesticides provide great economic benefits to human beings, they also pose a potential threat to human health. Pesticides, which are widely used, are important risk factors affecting human health. Cyfluthrin (Cy) is an important type II pyrethroid insecticide [1]. Because of its low toxicity and high efficiency, the fact that it leaves little residue and the wide range of pests it can control, it has been widely used in the agriculture, forestry, animal husbandry and fishery industries since its development in 1984 [2]. With the increasing use of Cy, the potential health effects of Cy residues on soil organisms and human beings have become a hot issue [3]. The accumulation of Cy in the body causes disorders of the nervous system, digestive system, reproductive system, cardiovascular system and immune system [4,5,6,7]. Moreover, a previous research group found that approximately 57% of the investigated vegetable greenhouse growers used dangerous concentrations of Cy, which were higher than those of other pyrethroid insecticides used [8].

Cy is somewhat soluble in fat. After entering the body, Cy can pass through the blood–brain barrier, accumulate in brain tissue and damage the human nervous system to varying degrees [9]. Some epidemiological investigations have also shown that long-term exposure to Cy and other pyrethroid pesticides can lead to a variety of nervous system disorders and even the precursor symptoms of neurodegenerative diseases [10,11]. Therefore, in this study, a rat model of nerve injury induced via Cy exposure was established to explore the relationship between Cy exposure and neurobehaviour, synaptic plasticity, the inflammatory response and adenosine A_2A_R expression in rats and the damaging effect of Cy exposure on the nervous system of Wistar rats.

## 2. Material and Method

### 2.1. Experimental Reagent and Dose Selection

Cy (c22h18cl2fno3, molecular weight 434.4, CAS NO.68359-37-5) was purchased from Dr. Company in the United Kingdom, and the purity was 99.52%. The solvent was a non-GMO corn oil. According to the relevant literature [12] and the results of previous experiments, the dosage groups were set as the solvent control group (corn oil), low-dose (6.25 mg/kg of Cy) group, medium-dose (12.5 mg/kg of Cy) group and high-dose (25 mg/kg of Cy) group.

### 2.2. Animals

Forty male SPF grade Wistar rats weighing approximately 270 g were purchased from Liaoning Changsheng Biotechnology Co., Ltd. (Shenyang, China) (licence No.: scxk (x) 20150001) and housed in the Animal Experiment Centre of Ningxia Medical University. The rats were allowed to adapt for one week and were housed at a room temperature of approximately 22 °C and relative humidity of approximately 60% on a normal day/night cycle. During the feeding period, they were allowed to freely drink and eat. The bedding was changed every three days.

**Figure 1:**
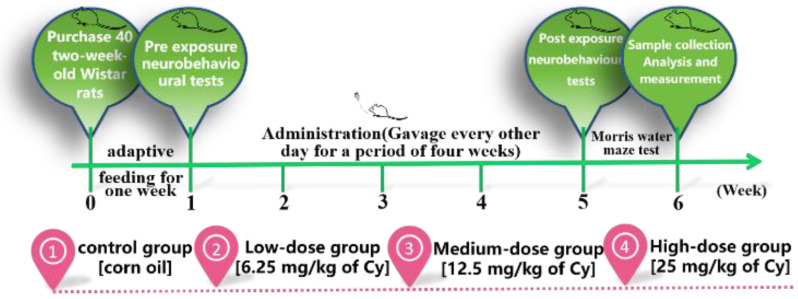
Experimental design schedule.

After one week of adaptation, the 40 Wistar rats were randomly divided into four groups with 10 rats in each group via the tail labelling method and a random number table generated by SPSS. The rats were weighed every other day, and the volume of the drug was selected according to the body weight measured on the previous day. The same concentration of the drug was administered to each animal via gavage, ensuring that the gavage volume did not exceed 4 mL per animal (a 16th gavage needle and 2.5 mL syringe were used). Each dose was administered between 8:00 am and 10:00 am, and the volume was adjusted every other day over four weeks. After the test, the rats were anaesthetized and sacrificed for follow-up experiments(Figure 1).

### 2.3. Tissue Sampling

Four randomly selected rats from each group were perfused and fixed with paraformaldehyde (Seville Company in China Wuhan) after anaesthesia, and then their brain tissue was quickly removed and fully fixed in paraformaldehyde. The hippocampal tissues of other rats were removed and frozen at −80 °C for electron microscopy. All animal protocols were performed in strict compliance with the relevant provisions of the People’s Republic of China on the use of experimental animals, and the experimental methods were approved by the ethics committee of Ningxia Medical University (No.: iacuc-nylac-2019-076).

### 2.4. Neurobehavioural Tests

#### 2.4.1. Open Field Experiment

The open field test was used to evaluate autonomous activity, exploratory behaviour and anxiety-like behaviour in a new environment. The experimental apparatus was composed of a grey-black square plastic box with a bottom area of 100 × 100 cm^2^ and a height of 60 cm. The bottom of the open field was divided into 16 square grids by black solid lines, and the middle 4 grids were defined as the central area. Artificial lighting was used, and the experiment was carried out in a quiet environment. Each rat was placed in the centre of the open field in turn and allowed to explore freely for five minutes, and relevant parameters were recorded. The method was carried out as previously described by Qin et al. [13].

#### 2.4.2. Novel Object Recognition Test

The novel object recognition test is a learning and memory test method based on the principle that rats have an innate tendency to explore new objects. This test is a validated method for studying recognition memory that can approximately simulate human learning and memory under free activity and be used to evaluate the formation of long-term or short-term memory in test animals by assessing their ability to identify changes in the shape and size of objects. The method of Ennaceur was carried out as previously described by Ennaceur et al. [14].

#### 2.4.3. Elevated plus Maze Test

The elevated plus maze is a widely used test of anxiety and curiosity. In this test, animals are presented with a new and different environment to assess their anxiety level and exploration [15]. The elevated plus maze consisted of a maze with a pair of relatively open arms (50 cm long × 10 cm wide), a pair of relatively closed arms (50 cm long × 10 cm wide × 40 cm high) and a central area (10 cm × 10 cm). The entire apparatus was grey-black, and it was made of non-reflective, good-quality medical-grade organic material. Under normal conditions, rats have a preference for small, dark spaces. However, they can exhibit exploratory behaviours and explore the open arms of the elevated plus maze. A decrease in open-arm activity indicates anxiety. The method of Knight was carried out as previously described by Knight et al. [16].

#### 2.4.4. Morris Water Maze Test

In the Morris water maze test, rats are forced to swim and find a platform hidden underwater. As a classical test for assessing spatial learning and memory, it is widely used in research on learning and memory, neurodegenerative diseases, hippocampal/extrahippocampal function, toxicology, preventive medicine, neuroethology and other fields. The Morris water maze is mainly used to test the ability of experimental animals to sense spatial position and direction (spatial positioning). There were two parts of the experiment: the positioning navigation phase and the spatial exploration phase. The experiment lasted for seven days and was divided into three stages. The method of Goudarzi was carried out as previously described by Goudarzi [17].

### 2.5. HE/Nissl Staining

For staining, tissue samples were collected, washed, dehydrated, cleared, sectioned, stained and mounted.

For HE staining, the sections were dewaxed and hydrated by soaking in xylene I for 15 min, xylene II for 10 min, absolute ethanol I for 5 min, absolute ethanol II for 5 min and 95%, 80% and 70% alcohol for 5 min each and rinsed with distilled water 3 times. Then, the sections were immersed in haematoxylin solution for 5 min, rinsed with running water, differentiated with hydrochloric acid and alcohol for 1 s and blued with distilled water. Next, the sections were immersed in eosin solution for 3 min and rinsed with running water. Finally, the sections were dehydrated in 75%, 85%, 95% ethanol and absolute ethanol I and II for 2 min each and soaked in xylene I and II for 4 min each. Neutral gum was used to mount the slides, and light microscopy was used to observe the tissue and capture images. The basic steps used for Nissl staining were the same as those used for HE staining.

### 2.6. Immunohistochemistry

Tissue samples were sectioned, dewaxed, hydrated and subjected to antigen repair. Then, a circle was drawn around the tissue with an immunohistochemical pen to identify the region of interest. The sections were incubated with 3% hydrogen peroxide at 37 °C for 15 min, rinsed with PBS, blocked, rinsed again with PBS three times for 5 min each and incubated with a horseradish peroxidase-labelled goat anti-rabbit IgG antibody. Then, they were incubated in a 37 °C oven for 20 min and rinsed with PBS three times for 5 min; each was incubated with haematoxylin solution for 5 min, rinsed with tap water, hydrochloric acid and alcohol, blued with distilled water, dehydrated, sealed and imaged.

### 2.7. Transmission Electron Microscopy

Coronal hippocampal tissue blocks were placed in a 2.5% glutaraldehyde fixation solution precooled at 4 °C for 30 min. The tissue blocks were cut into 3.1 × 2.0 × 1.0 mm pieces and fixed for 2 h. They were washed with buffer three times for 2 h each in a 4 °C refrigerator. The tissue blocks were then soaked in buffer and fixed in 1% osmic acid for 1 h, rinsed in buffer three times and dehydrated in 30%, 50%, 70%, 80%, 90%, 100% ethanol and 100% acetone for 10 min each. Then, they were embedded in epoxy resin, dried overnight in a 37 °C oven and polymerized in a 60 °C oven for 48 h. Uranium land lead double staining (2% uranyl acetate saturated alcohol solution and lead citrate, 15 min each) was performed, and the slices were dried overnight at room temperature. Images were collected under a transmission electron microscope for analysis.

### 2.8. Western Blot

Protein expression was measured according to the method of Jamal et al. [18] Briefly, hippocampal tissue was removed from a −80 °C ultralow temperature freezer and accurately weighed, and the weight of the tissue was recorded. The tissue was placed in a low-temperature grinding tube. Lysis reagent was prepared with PMSF solution at a ratio of 100:1, and the tissue was ground, placed on ice for 30 min, and centrifuged at 12,000 r/min for homogenization. The sample was centrifuged at 4 °C for 10 min, and the supernatant was collected. The total protein concentration was quantified using a BCA protein kit (Biyuntian) according to the manufacturer’s instructions. The protein samples were added to 10 times the volume of PBS in an EP tube heated at 100 °C for 10 min, denatured and stored at −20 °C for future use. The prepared samples (15 µL protein/lane) were electrophoresed on a 12% SDS–PAGE gel and electroblotted onto PVDF membranes (Thermo Company, Waltham, U.S.A.) and incubated with 3% BSA (Biopped Biotechnology Company, Beijing, China) on a shaking table for 1 h. After subsequent washing, the blots were incubated with primary antibody (Table 1) for 24 h at 4 °C followed by incubation with horseradish peroxidase-linked secondary antibody (anti-rabbit IgG, 1:2000) at room temperature for 60 min. After incubation, the secondary antibody was recovered according to the recommended procedure, and an appropriate amount of TBST (Shanghai double helix Biotechnology Co., Ltd., Shanghai, China) was added for elution.

### 2.9. Q-PCR

According to the method of Hong-qiang Chen [19], total RNA was extracted using a TRIzol reagent. The RNA (2 µg) was reverse transcribed with the GoScript™ Reverse Transcription System (Promega, Madison, WI, USA) according to the manufacturer’s instructions. Real-time qPCR was performed on a fluorescent quantitative PCR instrument (Bio-Rad, Hercules, CA, USA) using a fluorescent quantitative kit (Promega, USA). The sequences of the primers used for RT–PCR are shown in Table 2. The data were analyzed with respect to a calibration sample using the 2^−∆∆Ct^ method. All assays were conducted at least in triplicate.

### 2.10. ATP/Glu Test

Using the ATP testing kit provided by Solarbio Technology Co., Ltd. (Beijing, China), we determined the ATP content in a 96-well plate, and samples and standard reagents were added as instructed. The absorbance value was immediately measured at 340 nm as A1. The average OD value of each group was calculated. After incubation at 37 degrees for 30 min, the absorbance value was immediately measured at 340 nm as A2, and the average OD value of each group was calculated. All tests shall be made in triplicate. Calculate the ATP content according to the following company.

∆A_measurement_ = A2_measurement tube_ − A1_measurement tube_

∆A_standard_ = A2_standard_ − A1_standard_

ATP (μmol/g) = ∆A_measurement_ ÷ (∆A_standard_ ÷ C_standard solution concentration_) × V_sample volume_ ÷ W_sample mass_

Using the Glu testing kit provided by Solarbio Technology Co., Ltd. (Beijing, China), we determined the Glu content in a 96-well plate, and samples and standard reagents were added as instructed. The absorbance value was immediately measured at 340 nm as A1. The average OD value of each group was calculated. After incubation at 37 °C for 5 min, the absorbance value was immediately measured at 340 nm as A2, and the average OD value of each group was calculated. For each sample, the ∆A values were substituted into a standard curve fitting equation to determine the Glu (X). All tests were made in triplicate. We calculated the Glu content according to the following company.

Glu (μmol/g) = X ÷ sample mass

### 2.11. Statistical Analysis

SPSS 26.0 software was used to sort and analyze the data. The data are expressed as the $\bar{X}$ ± SD. ANOVA was used for comparisons among multiple samples; repeated measure ANOVA was used for the analysis of repeated measurement data; the Kruskal–Wallis test was used for nonparametric data; and Pearson correlation analysis was used for correlation analysis. The significance level was *p* ≤ 0.05. The grey values of the WB bands were analyzed by ImageJ 1.54, the fluorescence intensity was analyzed by Image-Pro Plus 6.0, and graphs were generated with GraphPad Prism 8.0.1.

## 3. Results

### 3.1. General Changes in Rats Exposed to Cy

**Figure 2:**
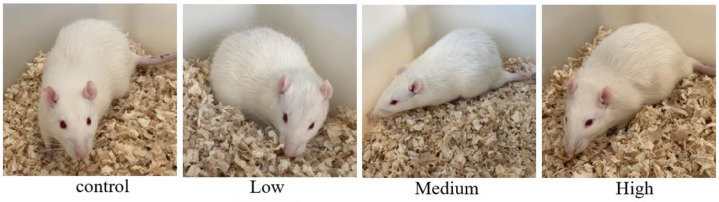
The state of rats exposed to different Cy doses.

**Figure 3:**
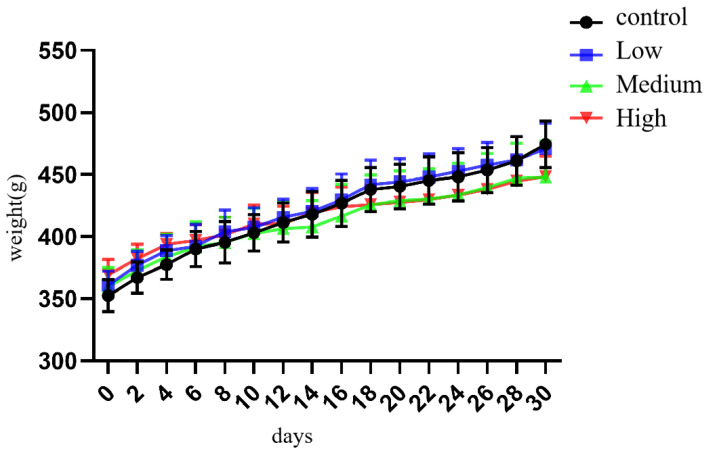
The weight changes in rats in each group.

Throughout the whole experimental process, the growth and development of rats in each group were not significantly abnormal. However, for observations during feeding, the resting state of rats in the middle- and high-dose exposure groups was higher than that of control rats, and the rats in the middle- and high-dose groups were more prone to irritability when subjected to intragastric administration and stimulation. As the exposure duration increased, the body weight of rats in each group showed an increasing trend. According to repeated measurements, there were differences in body weight grain on different exposure days (F = 103.128, *p* < 0.001). Body weight gain was significantly slowed in the medium- and high-dose groups in the middle of the exposure period, but there was no significant difference in body weight grain between the medium- and high-dose groups (f = 0.805, *p* > 0.05) (Figure 2 and Figure 3).

### 3.2. Changes in Learning and Memory Ability in Cy-Exposed Rats

#### 3.2.1. Open Field Experiment

**Figure 4:**
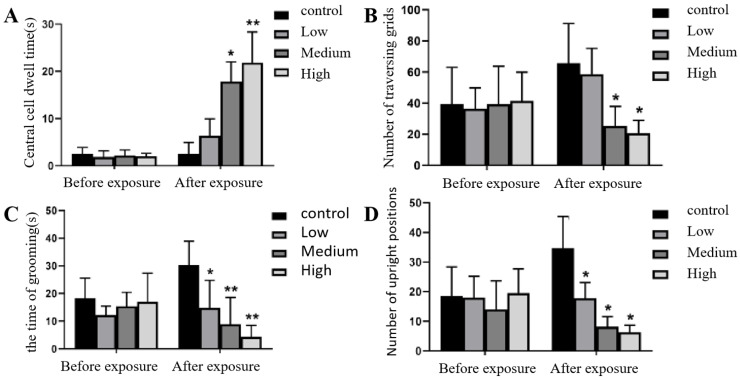
Changes in behaviour in the open field test before and after Cy exposure. (**A**) shows the time spent in the central zone by rats in each group before and after exposure; (**B**) shows the number of grid crossings before and after exposure; (**C**) shows the grooming time before and after exposure; (**D**) shows the number of rearings before and after exposure; compared with the control group, * *p* < 0.05, ** *p* < 0.001.

The results showed that in the open field test, before Cy exposure, there was no significant difference in the time spent in the central zone, the number of grid crossings, the grooming time or the number of rearings among the exposure group (*p* > 0.05). After Cy exposure, the time spent in the central zone was significantly increased in the middle- and high-dose Cy exposure groups compared with the control group (*p* < 0.05, *p* < 0.001), while the number of grid crossings in the middle- and high-dose Cy exposure groups was significantly decreased compared with that in the control group (*p* < 0.05). The grooming time and the number of rearings showed a gradual downwards trend in each exposure group compared with the control group, and the difference was statistically significant (*p* < 0.05) (Figure 4).

#### 3.2.2. Novel Object Recognition Test

**Figure 5:**
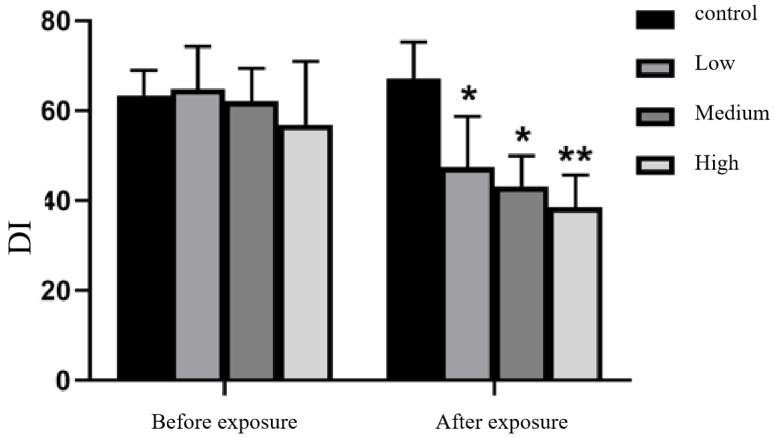
Changes in the DI of the novel object recognition test before and after Cy exposure Compared with the control group, * *p* < 0.05, ** *p* < 0.001.

The results showed that in the novel object recognition test, before Cy exposure, there was no significant difference in the novel object recognition index (DI) among the rats in the treatment groups and those in the control group (*p* > 0.05). The DI was significantly decreased in the low, medium and high Cy dose groups compared with the control group, statistically significantly (Figure 5).

#### 3.2.3. Elevated plus Maze Test

**Figure 6:**
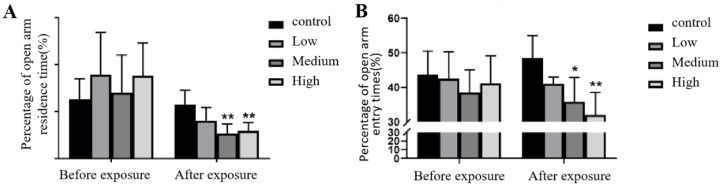
Changes in metrics in the elevated plus cross maze before and after Cy exposure. (**A**) shows the percentage of time spent in the open arms; (**B**) shows the percentage of open arm entries. Compared with the control group, * *p* < 0.05, ** *p* < 0.001.

The results showed that in the elevated plus maze test, there was no significant difference in the percentage of time spent in the open arms or the number of open arm entries among the groups before Cy exposure (*p* > 0.05). After Cy exposure, the percentage of time spent in the open arms and the number of open arm entries in the middle and high Cy dose groups significantly decreased compared with those of the rats in the control group, statistically significantly (Figure 6).

#### 3.2.4. Morris Water Maze Test

The Morris water maze is used to verify cognitive dysfunction in rodent models and evaluate the efficacy of drugs on cognitive function.

(1) Positioning navigation phase

**Figure 7:**
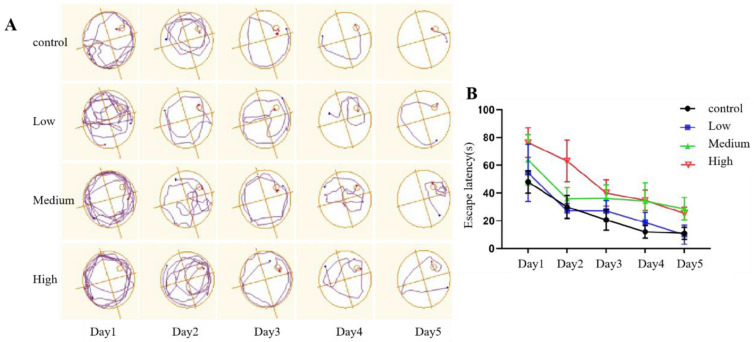
Comparison of exploratory behaviour and the escape latency. (**A**): Trajectories in the water maze. (**B**): Escape latency.

The escape latency was used to evaluate the spatial learning and memory of the rats. According to repeated-measures ANOVA, in the positioning navigation experiment, as the training time increased, the escape latency of rats in each group significantly decreased, and learning significantly increased (*p* < 0.001), indicating as the training period increased, the spatial learning and memory of rats in each group improved. Compared with that of rats in the control group, the escape latency of rats in the exposure groups was significantly prolonged in a dose-dependent manner (*p* < 0.001), indicating that Cy exposure may reduce the spatial learning and memory abilities of rats. However, there was no interaction between Cy exposure dose and training time (*p* > 0.05), indicating that training time had no effect on the change in escape latency or the change in the learning and memory abilities of rats induced by Cy exposure (Figure 7).

(2) Spatial exploration phase

**Figure 8:**
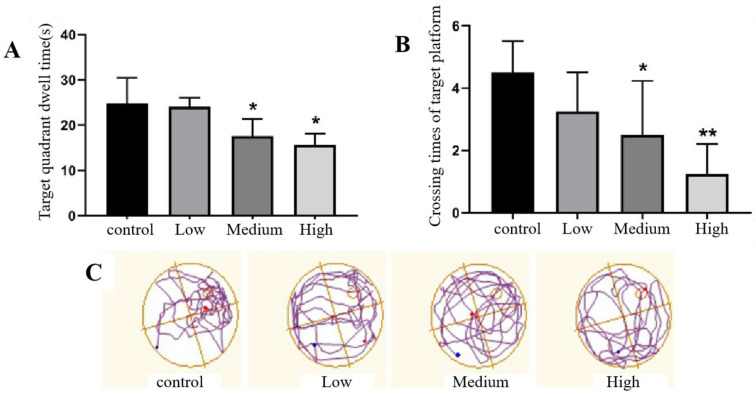
Comparison of the spatial exploration ability of rats exposed to Cy in the Morris water maze test. (**A**) shows the time spent in the target quadrant by the rats. (**B**) shows the number of platform crossings. (**C**) shows the trajectory of the rats. Compared with the control group, * *p* < 0.05, ** *p* < 0.001.

The spatial exploration of rats was assessed by calculating the spatial exploration index in the water maze. The experimental results showed that there was no significant difference in the time spent in the target quadrant or the number of target platform crossings in the low-dose Cy exposure group compared with the control group (*p* > 0.05), indicating that the effect of low-dose Cy exposure on the spatial exploration of rats was not significant. However, the time spent in the target quadrant and the number of target platform crossings was significantly decreased in the middle- and high-dose Cy exposure groups compared with the control group (*p* > 0.05), indicating that higher Cy dose reduced the spatial exploration ability of rats (Figure 8).

### 3.3. Hippocampal Neuron Injury in Rats Exposed to Cy

#### 3.3.1. HE Staining

**Figure 9:**
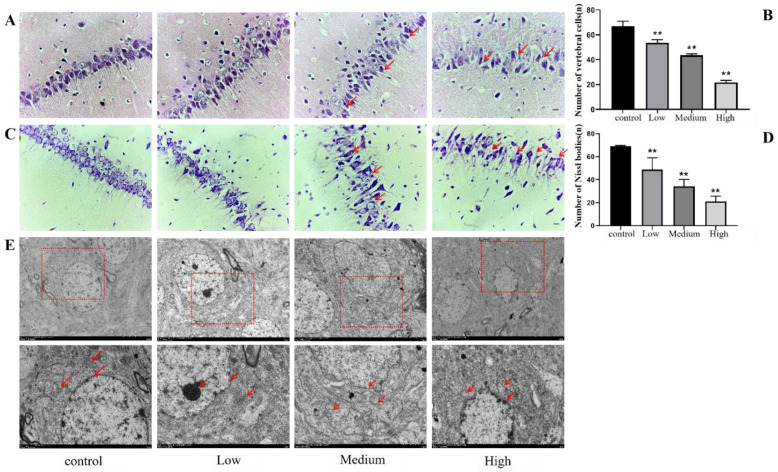
Injury to hippocampal neurons in rats exposed to Cy. (**A**): Comparison of HE-stained tissues (bar = 20 μm, 400 ×). (**B**): Statistical analysis of the number of vertebral cells. (**C**): Comparison of Nissl bodies in neurons (bar = 20 μm, 400 ×). (**D**): Statistical analysis of the number of Nissl bodies. (**E**): Morphological changes in hippocampal cells under an electron microscope. Bar = 5.0 μm, 1200×, bar=2.0 μm, 8000×. Compared with the control group, ** *p* < 0.001.

After the water maze test, whole brain sections were fixed in paraformaldehyde, embedded in paraffin and stained with HE to verify whether Cy exposure caused damage to neurons in the hippocampus of rats. The results showed that the cytoplasm of the hippocampal CA1 neurons was intensely stained in the different exposure groups, especially in the medium- and high-dose Cy exposure groups, compared with the control group. Moreover, neuronal cell bodies were smaller and degraded, the arrangement of neurons was loose and disordered, the structure of neurons was disordered, inclusions were swollen, the nuclei were pyknotic, and vacuolization was observed (Figure 9A). Moreover, the number of neurons in the Cy-exposed groups decreased significantly compared to that in the control group (*p* < 0.001) (Figure 9B), and the nuclei of some cells were completely degraded, resulting in the formation of ghost cells. In conclusion, as the Cy dose increased, the degree of neuronal damage in the rat hippocampus progressively increased, preliminarily verifying that Cy exposure causes morphological damage in the rat nervous system.

#### 3.3.2. Nissl Staining

After the water maze test, the whole brain was fixed with paraformaldehyde, embedded in paraffin and sectioned for Nissl staining. Nissl staining was used to detect Nissl bodies in nerve cells, which could further reveal the detrimental changes caused by Cy exposure to the morphology and number of neurons in the hippocampus. The results showed that, compared with the control group, the low-dose Cy group had only a small number of hyperchromatic nuclei and a decreased number of Nissl bodies, but the arrangement of the cells was relatively neat, and Nissl bodies were still visible in most of the neuronal envelopes. Compared with those in the control group, the hippocampal neurons in the middle- and high-dose Cy groups were mostly absent, disorderly and loosely arranged, while the plasma was intensely stained, and the number of Nissl bodies was significantly reduced. Microscopically, Nissl corpuscles were dark blue, the nuclei were pale blue, and the background was basically colourless (Figure 9C). By counting the number of intact Nissl bodies in rat hippocampal neurons, it was found that the number of Nissl bodies decreased significantly with increasing. Cy dose (*p* < 0.05) (Figure 9D), which further showed that Cy exposure had a damaging effect on rat hippocampal neurons.

#### 3.3.3. Electron Microscopy

To further observe the damage to hippocampal neurons in rats exposed to Cy, scanning electron microscope images were captured to observe ultrastructural changes in hippocampal neurons. The electron microscopy results showed that the cellular inclusions and nuclei in hippocampal neurons in the control group were complete and round, a double-layer nuclear membrane was clearly visible, and each organelle was clear and complete and in good condition, without any abnormalities. However, with increasing Cy dose, hippocampal neurons showed different degrees of damage, especially in the medium- and high-dose groups. The cell membrane and nuclear membrane of hippocampal neurons were severely shrunken and deformed, the double-layer nuclear membrane was blurred, the number of organelles was markedly reduced, the morphology of the organelles was abnormal, the mitochondrial ridge was disrupted, and lipid droplets were phagocytosed by lysosomes (Figure 9E). These findings further show that Cy also caused ultrastructural changes that could affect energy metabolism, signal transmission and other cellular functions in hippocampal neurons.

### 3.4. Impairment of Hippocampal Neuron Plasticity Induced by Cy Exposure in Rats

**Figure 10:**
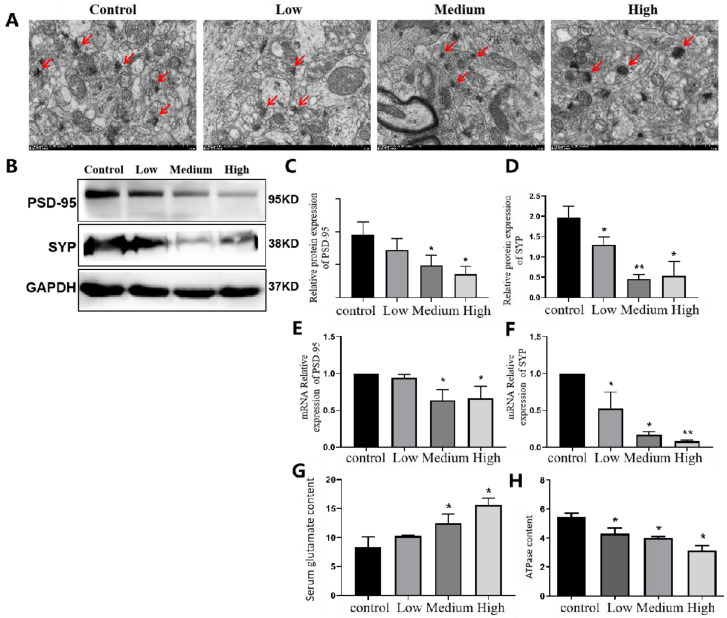
Comparison of synaptic Ji and PSD-95 and SYP protein and mRNA expression in the hippocampal neurons of Cy-exposed rats. (**A**): Electron microscopy results. Bar = 1.0 μm, 12,000×. (**B**): Western blot results for the different exposure groups. (**C**,**D**): Statistical analysis of PSD-95 and SYP protein expression. (**E**,**F**): Statistical analysis of the mRNA expression of PSD-95 and SYP. (**G**,**H**): Levels of glutamate and ATPase. Compared with the control group, * *p* < 0.05, ** *p* < 0.001.

The electron microscopy results showed that in the control group, the number of synapses in hippocampal neurons was high, the synaptic structure was clear and complete, the presynaptic membrane, synaptic vesicles and postsynaptic modules were clearly visible, and the synaptic gap was moderate. As the Cy dose increased, the number of synapses in hippocampal neurons gradually decreased, and the synaptic structure became abnormal, mainly manifested in the disappearance of synaptosomes and the degradation of the presynaptic and postsynaptic membranes. The synaptic space became narrower, the synaptic vesicles decreased in number or disappeared, and the overall synaptic morphology showed ground-glass opacity. The area indicated by the synapses was opaque. The arrow shows morphological changes in synaptic vesicles at the presynaptic membrane terminals (Figure 10A).

Western blot analysis of the expression of the synaptic marker protein PSD-95 and synaptic vesicular protein SYP showed that, compared with that in the control group, the expression of PSD-95 in the middle- and high-dose Cy groups was significantly lower (*p* < 0.05), while the expression of the synaptic vesicular protein SYP in the hippocampus of rats in all exposed groups showed a significant decrease compared with that in the control group(*p* < 0.05). The changes in PSD-95 and SYP mRNA expression were consistent with the changes in their protein expression (Figure 10E,F). The results of the ATP and the Glu test showed that compared with that in the control group, the ATPase level in the hippocampus decreased with increasing Cy dose (*p* < 0.05) (Figure 10H). Compared with that in the control group, the level of the neurotransmitter Glu in the hippocampus significantly increased with increasing Cy dose (*p* < 0.05) (Figure 10G).

In conclusion, Cy exposure can impair hippocampal neuron synaptic plasticity to a certain degree by altering morphology and the expression of key synaptic proteins and has a negative impact on neurotransmitter release.

### 3.5. Changes in the Levels of Inflammatory Factors and A_2A_R-Related Factors in the Neurons of Rats Exposed to Cy

#### 3.5.1. Expression of Inflammatory Factors in the Hippocampus of Cy-Exposed Rats

**Figure 11:**
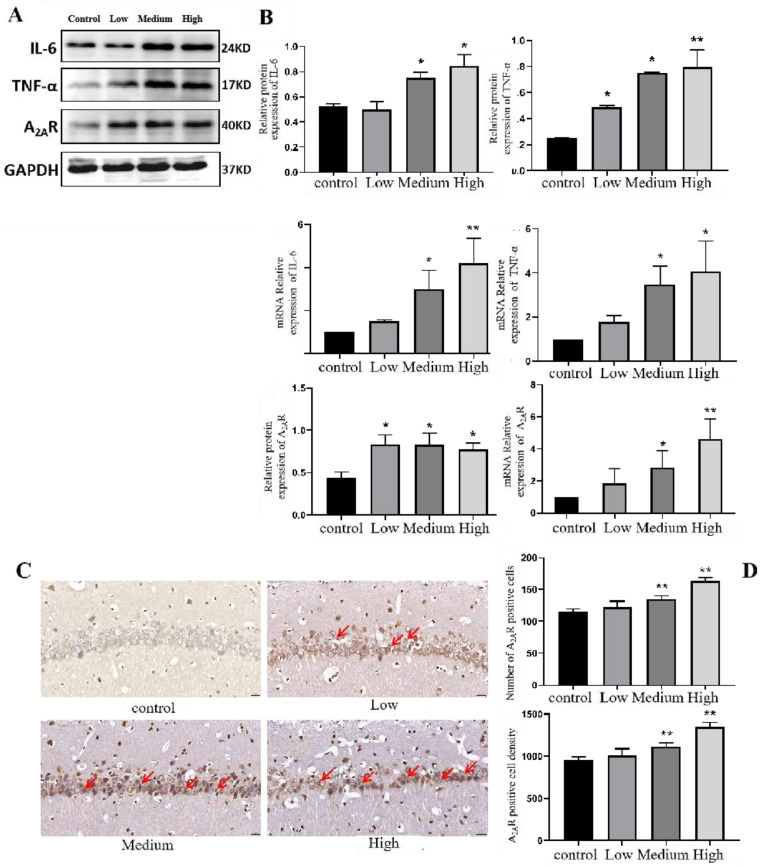
Comparison of the expression levels and immunohistochemical staining of IL-6, THF-α and A_2A_R proteins and mRNA levels in Cy-exposed rats. (**A**): Western blotting results. Bar = 20 μm, 400×. (**B**): Statistical analysis of IL-6, THF-α, A_2A_R protein and mRNA expression. (**C**,**D**): Immunohistochemistry results. Compared with the control group, * *p* < 0.05, ** *p* < 0.001.

The protein expression of inflammation-related factors in the hippocampus was measured to explore the further damaging effect of Cy exposure. The Western blot results showed that compared with the control treatment, the medium and high doses of Cy significantly upregulated the inflammatory factor IL-6 (*p* < 0.05). Compared with the control treatment, the low, medium and high doses of Cy significantly upregulated the inflammatory factor TNF-α (*p* < 0.05) (Figure 11B).

Moreover, the mRNA expression of inflammatory factors was measured by Q-PCR. The results showed that compared with the control treatment, the medium and high doses of Cy could significantly upregulate the inflammatory factors IL-6 and TNF-α (*p* < 0.05) (Figure 11B).

#### 3.5.2. Expression of A_2A_R in the Hippocampus of Cy-Exposed Rats

The correlation between the degree of hippocampal tissue damage caused by Cy exposure and the expression of A_2A_R was explored, and immunohistochemical staining of hippocampal tissue from rats in each group showed that A_2A_R was expressed in the cytoplasm of neurons. The red arrows in the figure indicate typical A_2A_R-positive cells (Figure 11C). Compared with that in the control group, the number of A_2A_R-positive cells was significantly increased in the middle- and high-dose Cy groups (*p* < 0.001) (Figure 11D). Furthermore, the Q-PCR results showed that compared with that in the control group, the expression of A_2A_R mRNA in the medium- and high-dose Cy exposure groups was significantly downregulated (*p* < 0.05) (Figure 11B).

In conclusion, Cy exposure can lead to abnormal expression of A_2A_R and inflammatory factors in rat hippocampal tissue.

### 3.6. Correlation Analysis of A_2A_R with Neurobehavioural Indices, Inflammatory Factors Levels and Synaptic Plasticity-Related Factor Levels

The results of the Pearson correlation analysis showed that the expression of A_2A_R mRNA was positively correlated with the time spent in the central zone in the open field test and the average escape latency in the water maze test (*p* < 0.001) and negatively correlated with other neurobehavioural indices (*p* < 0.05). Moreover, the expression of A_2A_R mRNA was strongly correlation with indices in the open field, novel object recognition and water maze test. These three tests were mainly used to assess recognition memory, spatial memory and spatial exploration. In addition, the expression of A_2A_R mRNA was positively correlated with the expression of inflammatory factors (*p* < 0.05) and negatively correlated with the expression of synaptic plasticity-related factors (*p* < 0.05)(Table 3).

## 4. Discussion

Cy is an important type II pyrethroid pesticide. Because its molecular structure contains a benzene ring, Cy is soluble in fat, and research has shown that when Cy enters the body, it can pass through the blood–brain barrier and accumulate in brain tissue [9]. In this study, there was no significant difference in the weight of rats among the group. However, compared with those in the control group, the rats in the medium- and high-dose exposure groups were more sluggish and were more prone to irritability during intragastric administration and stimulation.

### 4.1. Cy Exposure Can Cause Neurobehavioural Changes in Rats

Normal behavioural function is the embodiment of the complete morphological structure and physiological function of the nervous system. Damage to the nervous system can be directly reflected by abnormalities in neurobehavioural function [20]. Therefore, the most direct method to evaluate the damaging effect of neurotoxins on the nervous system is to perform neurobehaviour tests in animals. Studies have also shown that neurobehavioural abnormalities caused by neurotoxins generally occur earlier than biochemical and pathological changes, so neurobehavioural tests are also more sensitive than biochemical tests and pathological analysis [21]. In this study, four neurobehavioural experiments were conducted to evaluate the degree of neurobehavioural impairment in rats exposed to Cy.

The open field experiment is used to assess the exploration ability and spatial cognition of experimental animals. It is used to evaluate the exploratory behaviour and anxiety level of rats in unfamiliar environments [22]. The results showed that there was no significant difference in any of the indices in the open field test among the group before exposure, indicating that the animals were indeed randomly allocated to the different groups. The number of grid crossings, grooming time and number of rearings decreased to varying degrees in the Cy-exposed group, while the time spent in the central zone was significantly prolonged. These changes were dose-dependent, indicating that as the Cy dose increased, the exploration of rats in the new environment decreased. Furthermore, excitement and exploration ability decreased. Some studies have also shown that when the learning and memory of rats are abnormal, their exploratory behaviour and activity decrease [23].

The novel object recognition test is used to assess the exploration and recognition of novel objects by rats [14]. The results showed that there was no difference in the discrimination index of rats among the group before exposure, but as the Cy dose and exposure duration increased, the ability of the rats to recognize the novel objects decreased. The effect was especially seen in the high-dose Cy exposure group. This finding indicated that Cy exposure reduced the ability of rats to recognize novel objects and impaired their memory and is consistent with the results of Hughes, MF [24].

The elevated plus maze test is a behavioural test used to evaluate the exploratory behaviour and anxiety level of rats [16,25]. The results of this study showed that there was no difference in the number of entries into or the time spent in the open arms before exposure, but after exposure, the number of entries into and time spent in the open arms by rats in the medium- and high-dose groups was significantly decreased, indicating that the exploratory behaviour of the rats decreased, and their level of anxiety increased. Moreover, some studies have shown that reductions in the number of entries into and the time spent in the open arms by rats in the elevated plus maze may be related to cognitive impairment and abnormal neurotransmitter release [25].

The Morris water maze test is a classic behavioural experiment used to test the spatial learning and memory of rats. It is one of the most commonly used methods to evaluate the impairment of learning and memory ability in rats [17,26]. It is a practical, reliable and easy-to-implement method designed by Morris in 1981 [27]. In this experiment, the rats were trained for 5 days after Cy exposure. The results showed that the escape latency of rats in each group was shortened via continuous intensive training. However, a longitudinal comparison of the escape latency of rats in different groups showed that the escape latency also increased with increasing Cy dose, with Cy having a dose-dependent effect on the escape latency, indicating that Cy exposure reduced the spatial learning ability of rats. On the sixth day of training, the spatial exploration test was carried out. The results showed that the number of platform crossings and the time spent in the target quadrant in the medium- and high-dose exposure groups were significantly shorter than those in the control group, indicating that Cy impaired not only the spatial learning ability but also the short-term spatial memory ability of the rats, exerting a neurotoxic effect. This finding is consistent with a previous study by Syed F [28]. Since the hippocampus is the main brain region responsible for long-term and short-term memory and learning [29], it can be speculated that Cy exposure may damage the hippocampus of rats, thus impairing the learning and memory ability and causing nervous system diseases.

### 4.2. Cy Exposure Can Cause Morphological Changes in the Rat Hippocampus

Changes in function and behaviour may be accompanied by changes in tissue morphology. HE staining can be used to intuitively evaluate the degree to which toxic substances alter tissue morphology. Therefore, HE staining was used to observe the changes in hippocampal morphology after Cy exposure in this study. The results of HE staining showed that as the Cy dose increased, the cells in the hippocampal CA1 area exhibited vacuolization and pyknosis, their arrangement was disordered, and they were lost, with the high-dose exposure group showing the most marked changes, which further indicated that Cy exposure seriously disrupted the morphology of the rat hippocampus. To further observe neuronal damage, Nissl staining was used to assess the distribution and morphology of Nissl bodies [30]. The Nissl bodies are the main structures responsible for protein synthesis and can balance the levels of various neurotransmitters in the brain and maintain the stability of neuronal excitability. Nissl body damage is regarded as an important sign of neuronal damage [31]. The Nissl staining results showed that compared with the control group, the medium- and high-dose exposure groups exhibited loss and disordered and loose arrangement of hippocampal neurons, intense cytoplasmic staining and a reduction in the number of Nissl bodies. This confirmed that Cy exposure can cause dissolution and vacuolization of Nissl bodies in hippocampal neurons and damage to neurons. In addition, the transmission electron microscopy results show that as the Cy dose increased, the cell membrane and nuclear membrane of hippocampal neurons became severely shrunken and deformed, the double-layer nuclear membrane was blurred, the morphology of organelles was severely disrupted, and the number of organelles was markedly decreased. Moreover, mitochondrial ridge fragmentation and lysosome phagocytosis of lipid droplet vesicles were observed. Mitochondrial damage seriously affects energy metabolism and information transmission in cells [32], and nuclear pyknosis and nuclear membrane damage further affect the replication of DNA and the normal function of cells, resulting in cellular dysfunction, inflammation, apoptosis and other changes.

In conclusion, Cy exposure irreversibly impairs the function of organelles and nuclei and damages the morphology of cells, which may seriously affect the normal function of cells.

Cy exposure can cause abnormal synaptic plasticity and neurotransmitter release in the rat hippocampus. The brain consumes a large amount of energy, and energy metabolism in the brain is of great importance for its normal function, the transmission of information, and learning and memory [33]. Ultrastructure analysis revealed that the mitochondria of hippocampal neurons were seriously damaged, and a decrease in ATP levels, as the “currency” for intracellular energy transfer, could directly explain the impairment of cellular energy metabolism. Therefore, we determined the levels of ATPase in hippocampal tissue. The results showed that the level of ATPase in hippocampal tissue decreased in a dose-dependent manner, further indicating that Cy exposure had an impact on the morphology of hippocampal neurons. Moreover, Li Qingqing et al. found that changes in hippocampal structure and function are related to synaptic plasticity, which is closely related to learning and memory in rats [34,35,36]. Synaptic plasticity is one of the most basic and important functions of the brain. It underlies the ability to perceive, evaluate and store complex information. Moreover, it can allow adaptive responses to related stimuli, such as disordered energy metabolism, inflammation and apoptosis [37]. Synaptic plasticity includes changes to synaptic structure and function, that is, alterations in synaptic transmission efficiency and synaptic morphology [38]. This paper mainly focused on changes in synaptic structure. The transmission electron microscopy results showed that as the Cy dose increased, the number of synapses in hippocampal neurons gradually decreased and synaptic structure changed; this mainly manifested as the disappearance of synaptosomes, the blurring of the presynaptic and postsynaptic membranes, the narrowing of the synaptic gap, a reduction in the number or disappearance of synaptic vesicles and opacity of synapses. This finding further confirms that changes in neuronal cell morphology are accompanied by changes in neuronal synapse morphology and function [39]. Then, we determined the protein and mRNA expression of key proteins in synaptic plasticity, namely, postsynaptic density (PSD-95) and synaptophysin (SYP). The results showed that the expression of PSD-95 was significantly downregulated in the middle- and high-dose Cy exposure groups compared with the control group, while the expression of the SYP protein in the hippocampus of rats in the exposure group showed a downwards trend compared with the control group. The results of Q-PCR were consistent with those of Western blotting. PSD-95 is an important postsynaptic density protein. It plays key regulatory roles in the transmission of information between synapses, synaptic plasticity, the formation of synapses and the development of excitatory synapses [40,41]. A decrease in the expression level of PSD-95 directly reflects synaptic plasticity and the normal function of synapses. SYP, a vesicular membrane protein located at the end of axons, can be used to assess the number and distribution of synapses [42]. PSD-95 and SYP are not only markers of synaptic membranes but also of synaptic plasticity, as they are related to the formation and maturation of synapses. The loss of synapses may result in decreased secretion of PSD-95 and SYP [43]. After the structure of synapses is disrupted and the synapse number is decreased, the release of neurotransmitters, one of the important functions of synapses, is affected. Abnormal release of neurotransmitters may impair intercellular communication. Previous studies also found that Cy exposure can cause abnormal release of neurotransmitters in rats [44]. In this study, it was found that compared with the control group, the release of Glu in the medium- and high-dose groups showed a significant upwards trend. The release of a large amount of Glu can cause overexcitation of neurons and eventually cause damage [45]. Injury causes a large amount of Na^+^ and Cl^−^ to flow into the cells, causing neuronal swelling and necrosis. Moreover, Glu can act on glutamate receptors on the postsynaptic membrane, resulting in the opening of receptor-dependent Ca^2+^ channels, the influx of a large amount of Ca^2+^, the release of a large amount of Ca^2+^ from the endoplasmic reticulum, and severe overload of intracellular Ca^2+^ leading to neuronal necrosis. In conclusion, Cy exposure can cause changes in the morphology and structure of hippocampal neurons, impairing synaptic plasticity between neuronal cells, affecting the release of neurotransmitters, and disrupting cell morphology and function.

### 4.3. Cy Exposure Can Cause Abnormal Changes in the Inflammatory Response and Adenosine A_2A_R Expression in the Rat Hippocampus

Some studies have shown that damage to hippocampal neurons can be accompanied by hippocampal inflammation [46,47,48]. TNF-α can induce nerve injury by mediating neuroinflammation and promoting T cells to produce various inflammatory factors [49]. IL-6 has a wide range of biological activities. Specifically, it can regulate the immune response and promote B cells precursors to become antibody-producing cells, which is of great importance for reducing inflammation, neuronal degeneration and necrosis [50]. In this study, the expression of two important inflammatory factors, TNF-α and IL-6, in the hippocampus was determined. Compared with the control group, TNF-α and IL-6 protein and mRNA levels were significantly increasing in the exposure groups, suggesting that Cy is likely to induce an inflammatory response in the rat hippocampus, which is basically consistent with the results of Shang Jiaqi [51]. Studies have reported that A_2A_R can mediate a variety of physiological and pathological processes, including apoptosis, inflammation and synaptic plasticity [52,53]. Chen Jiangfan found that many A_2A_R-positive neurons can be seen in brain tissue after cerebral ischaemia, which confirms that A_2A_R is closely related to cerebral ischaemic injury [54]. Furthermore, some scholars have also found that inhibiting the expression of A_2A_R can alleviate brain tissue damage and that this protective effect is accompanied by a reduction in TNF-α levels in brain tissue. The downregulation of inflammatory factors, including IL-6, can reduce damage to neurons in the brain [55]. The results also showed that the protein and mRNA expression of A_2A_R in Cy-exposed rats, especially those in the middle- and high-dose groups, was significantly higher than that in control rats. Moreover, Pearson correlation analysis showed that there was a close relationship between the expression of A_2A_R mRNA and various neurobehavioural indicators and that the expression of A_2A_R mRNA was positively correlated with the levels of inflammatory factors and apoptosis-related factors. However, A_2A_R mRNA expression was negatively correlated with the levels of synaptic plasticity-related factors, which further suggests that A_2A_R may be involved in neuronal injury in the hippocampus induced by Cy exposure, but the specific mechanism needs to be further explored.

## 5. Conclusions

The tissue morphology and general condition of Cy-exposed rats were not significantly different from those of the control rats. However, neurobehavioural tests showed that learning and memory ability became impaired, exploratory behaviour decreased, and anxiety-like behaviours increased as the Cy dose increased.

Pathological analysis showed that the middle and high doses of Cy caused deformation and reduced the number of hippocampal pyramid cells, disrupted the arrangement of these cells, decreased the Nissl body number, caused pyknosis of hippocampal neuron nuclei and severely damaged organelles, indicating that Cy exposure at these doses may cause hippocampal tissue damage in rats.

In this study, as the Cy dose increased, the structural changes in hippocampal synapses became more obvious, with the synaptic gap being blurred and the numbers of synaptic vesicles and synapses being decreased. Furthermore, the expression of the key synaptic proteins PSD-95 and SYP decreased in a dose-dependent manner, indicating that Cy caused synaptic damage.

In this study, medium and high doses of Cy upregulated the expression of A_2A_R in hippocampal tissue. Moreover, it increased the protein expression levels of inflammatory factors and apoptosis-related factors in a dose-dependent manner, and there was a correlation between the expression of A_2A_R mRNA and neurobehavioural indicators, inflammatory factors levels and synaptic plasticity-related factor levels, suggesting that Cy may cause nerve damage in rats and that this effect may be closely related to A_2A_R.

## Figures and Tables

**Table 1:** Primary Antibodies.

Antibody Name	Company
Rabbit Anti-A_2A_R (ab3461)	Abcam (Cambridge, UK)
Rabbit Anti-PSD-95 (ab76115)	Abcam (Cambridge, UK)
Rabbit Anti-SYP (ab32127)	Abcam (Cambridge, UK)
Rabbit Anti-IL-6	Affinity (Boston, MA, USA)
Rabbit Anti-TNF-α	Affinity (Boston, MA, USA)
Rabbit Anti-GAPDH	Bioss (Beijing, China)

**Table 2:** Primer sequences.

	Forward Primer	Reverse Primer	Species
A_2A_R	GAAAGACGGGAACTCCACGAAGAC	GGCAGTAACACGAACGCAAAGAAG	Rat
PSD-95	TCCAGTCTGTGCGAGAGGTAGC	GGACGGATGAAGATGGCGATGG	Rat
SYP	GCTGTGTTTGCCTTCCTCTACTC	TGATAATGTTCTCTGGGTCCGTG	Rat
IL-6	ACTTCCAGCCAGTTGCCTTCTTG	TGGTCTGTTGTGGGTGGTATCCTC	Rat
TNF-α	AAAGGACACCATGAGCACGGAAAG	CGCCACGAGCAGGAATGAGAAG	Rat

**Table 3:** Correlation analysis between A_2A_R mRNA expression and related indicators.

Experimental Category	Indicators	Pearson Correlation Coefficient *r*	*p*
Open field test			
	Time spent in the central zone	0.7154	<0.001
	Grooming time	−0.6354	0.001
	Number of rearings	−0.4381	0.032
	Number of grid crossings	−0.5682	0.004
Novel object recognition test			
	Discrimination index	−0.6896	<0.001
Elevated plus maze test			
	Time spent in the open arms	−0.375	0.071
	Number of open-arm entries	−0.4633	0.023
Water maze test			
	Average escape latency	0.7000	<0.001
	Time spent in the target quadrant	−0.6044	0.002
	Number of platform crossings	−0.6364	0.001
Inflammation-related factors			
	IL-6	0.8612	<0.001
	TNF	0.8433	0.001
Synaptic plasticity-related factors			
	PSD-95	−0.6377	0.026
	SYP	−0.7935	0.002

## Data Availability

All data included in this study are available upon request via contact with the corresponding author.

## Abbreviations

The following abbreviations are used in this manuscript:

Cy	Cyfluthrin
A_2A_R	Adenosine2a receptor
ATP	Adenosine tri phosphate
Glu	Glutamate
Q-PCR	Real-time Quantitative PCR
HE	Hematoxylin-eosin
ANOVA	Analysis of variance
